# The Impact of Dupilumab on Work Productivity and Emotional Health in CRSwNP: A Multicentric Study in Northeast Italy

**DOI:** 10.3390/jpm14050468

**Published:** 2024-04-28

**Authors:** Giancarlo Ottaviano, Giuseppe Roccuzzo, Lucia Lora, Elisabetta Bison, Enrico Tosin, Leonardo Calvanese, Walter Cestaro, Luca Giovanni Locatello, Fabrizio Corlianò, Marco Stellin, Maria Baldovin, Maria Vittoria Trimarchi, Anna Giulia Bertocco, Marco Maculan, Bruno Scarpa, Tommaso Saccardo, Piero Nicolai

**Affiliations:** 1 Department of Neurosciences DNS, Otolaryngology Section, University of Padova, 35122 Padova, Italy; giuseppe.roccuzzo@studenti.unipd.it (G.R.);; 2 ENT Department, Ospedale dell’Angelo, ULSS 3 Serenissima, Mestre, 30174 Venezia, Italy; 3 ENT Department, Hospital of Cittadella, 35013 Padova, Italy; 4 ENT Department, Hospital of Rovigo, 45100 Rovigo, Italy; 5 ENT Unit, Department of Surgery, Ospedali Riuniti Padova Sud, 35043 Monselice, Italy; 6 ENT Consultant, Head and Neck Department, ULSS 2 Marca Trevigiana, 31100 Treviso, Italy; walter.cestaro@aulss2.veneto.it; 7 Department of Otorhinolaryngology, University Hospital “Santa Maria Della Misericordia”, Azienda Sanitaria Universitaria Friuli Centrale (ASUFC), 33100 Udine, Italy; lucagiovanni.locatello@asufc.sanita.fvg.it; 8 Department of Otolaryngology Head and Neck Surgery, San Bassiano Hospital, Via Dei Lotti 40, 36061 Bassano Del Grappa, Italy; 9 Department of Neurosciences, Section of Otolaryngology, University of Padova, 31100 Treviso, Italy; marco.stellin@aulss2.veneto.it; 10 ENT Unit, Ospedale San Martino, Belluno, ULSS1 Dolomiti, 32100 Belluno, Italy; 11 Department of Statistical Sciences, University of Padova, 35122 Padova, Italy; scarpa@stat.unipd.it

**Keywords:** Dupilumab, Qol, emotion, function, SNOT-22, productivity, concentration, fatigue, sadness, embarrassment, CRSwNP

## Abstract

Chronic rhinosinusitis with nasal polyps (CRSwNP) in the severe forms is associated with a poor quality of life. Dupilumab has been suggested as an add-on treatment option for severe CRSwNP. Severe CRSwNP patients treated with Dupilumab in different rhinological units were considered for this study via their evaluation at the baseline at first and the consequential follow-up at 6-, 12-, and 24 months from the first administration. At baseline (T0) and at each follow-up, patients underwent NPS, Sinonasal Outcome Test (SNOT)-22, Visual Analogue Scale (VAS) for smell, and Sniffin’ sticks identification test (SSIT). The SNOT-22 domains for function and emotion were also analysed separately. Two hundred and seventeen patients with at least 6 months of follow-up were included. All parameters have improved during treatment (*p* < 0.0001). Noticeably, both the function and emotion SNOT-22 domains have improved within 6 months of treatment and have continued to progress during every interval within 12 months from the baseline, positively influencing patients’ emotivity and augmenting their social and economic performances. Dupilumab improves the QoL of CRSwNP patients with good effects on the reported productivity and emotional health. Clinicians should pay attention to these two aspects when dealing with patients affected by severe CRSwNP.

## 1. Introduction

Chronic rhinosinusitis (CRS) is a prolonged inflammation of the nasal cavity and sinus mucosa lasting more than 12 weeks. CRS has traditionally been classified into either with nasal polyps (CRSwNP) or without nasal polyps (CRSsNP) [1]. The former accounts for approximately 30% of CRS cases [2] and has a higher disease burden than CRSsNP [1]. CRSwNP is a multifactorial disease that not only can be associated with genetic disorders, immunodeficiency, anatomical abnormalities, and chronic osteomyelitis, but it can also be influenced by exposure to environmental factors such as air pollution, smoke, allergens, viruses, bacteria, and fungi [3]. It has been recognised that the most common endotype of CRSwNP in the West Countries is type 2 inflammation [1,2]. CRSwNP is a common health condition in the Western world, with an estimated prevalence of approximately 3% [1,4]. Consistent with most of the Western Countries, Italy reports the majority of CRSwNP cases being affected by a type 2 inflammation with elevated levels of interleukin (IL)-4, IL-5, and IL-13, as well as a remarkable presence of eosinophils, type 2 innate lymphoid cells, macrophages, and mast cells [2,5,6,7]. As a chronic inflammatory disease, CRSwNP significantly affects wellbeing and social function, de facto associating CRSwNP with an increased risk of depression and social dysfunction [8], especially among young adults and middle-aged populations [1]. Similarly important is the extensively studied association between CRSwNP and sleep disturbance [9], which is linked to cardiovascular disease and reduced quality of life [10]. Of note, the impact of CRSwNP on quality of life (QoL) appears to be equivalent to chronic obstructive pulmonary diseases, congestive heart failure, and diabetes [11,12].

With the introduction of targeted biologic molecules in the management of severe CRSwNP, patients’ QoL has substantially improved [13]. Dupilumab, a human monoclonal antibody of the immunoglobulin G4 subclass able to block IL-4 and IL-13 signalling by specifically binding to the IL-4 receptor α subunit [9], has been demonstrated to be effective in reducing nasal polyps’ volume and improving the QoL of patients affected by severe CRSwNP [14], including older patients [15]. Being IL-4 and IL-13 relatively upstream players in the inflammatory cascade, these ILs are pivotal in the pathogenesis of both CRSwNP and asthma. At the moment, Dupilumab is approved for the treatment of moderate-to-severe atopic dermatitis, moderate-to-severe asthma with evidence of type 2 inflammation, eosinophilic esophagitis, prurigo nodularis and for the treatment of severe CRSwNP [9].

Both nasal obstruction and hypo-anosmia are the main symptoms reported by CRSwNP patients. In particular, more than 90% of CRSwNP patients complain of nasal blockage [16], while olfaction is impaired in about 83–91% of patients with CRSwNP [17], where the majority is anosmic [18].

The aim of the present multicentric study is to evaluate the effects of Dupilumab in patients with severe uncontrolled CRSwNP on wellbeing, with a special focus on productivity and improvement of emotion for patients residing in the region of Triveneto, one of the most industrialised areas of Italy [19,20].

## 2. Materials and Methods

### 2.1. Population

This is a no-profit, observational, retrospective, multicentric study. Study participants were recruited in the rhinological units of 10 different hospitals in Triveneto.

Inclusion criteria were defined as follows: (1) age ≥ 18 years; (2) diagnosis of severe chronic rhinosinusitis with nasal polyps (CRSwNP), defined by a nasal polyp score (NPS) ≥ 5 and/or a Sinonasal Outcome Tests-22 (SNOT-22) ≥ 50, with inadequate symptoms control despite intranasal corticosteroids (INCS) use, receiving at least two cycles of systemic corticosteroid in the last year and/or having undergone one or more sinonasal surgeries [(endoscopic sinus surgery (ESS)]; (3) at least 6 months of biological therapy administration (Dupilumab 300 mg, one subcutaneous injection every 15 days) indicated specifically for severe CRSwNP treatment.

A total of 217 patients were included in this study, recruited from February 2021 to September 2023: 73 patients from the Rhinological Unit of Padova University Hospital, 42 patients from the ENT Unit of Venezia Mestre “dell’Angelo” Hospital, 27 patients from the ENT Unit of Cittadella Hospital, 19 patients from the ENT Unit of Rovigo “Santa Maria della Misericordia” Hospital, 12 patients from the ENT Unit of Schiavonia “Madre Teresa di Calcutta” Hospital, 10 patients from the ENT Unit of Udine “Santa Maria della Misericordia” Hospital, 10 patients from the ENT Rhinological Unit of Montebelluna “San Valentino” Hospital, 8 patients from the ENT Unit of Treviso “Ca’ Foncello” Hospital, 8 patients from the ENT units of BellunoHospitals, and 8 patients from the ENT Unit of Bassano del Grappa “San Bassiano” Hospital. All patients underwent Dupilumab 300 mg, subcutaneous injection every 15 days, added to INCS as conventional treatment [21].

We collected data at baseline (before starting the biological treatment) (T0) and at the subsequent follow-up visits [6 months (T1), 12 months (T2), and 24 months (T3)]. During each visit patients’ QoL was assessed using the Sinonasal Outcome Test 22 (SNOT 22) [22], administered during each visit. SNOT 22 questions were therefore categorised into 5 distinct domains, namely nasal, ear/facial, sleep, function, and emotion [23]. Nasal obstruction and impairment of the sense of smell were also measured subjectively by means of the Visual Analogue Scale (VAS) for nasal obstruction (NO), smell, rhinorrhea and facial pain by 4 centres (Padova, Mestre, Schiavonia, Udine, Italy) for a total of 137 patients. Being more than half of the total population, we decided to include these data for the purpose of the analyses [6]. In addition to the subjective olfactory evaluation (VAS smell), olfaction was objectively assessed by means of SSIT (16 odours) (Burghart Messtechnik GmbH, Holm) [24,25]. Following Gevaert et al. [26], nasal polyps’ extension and severity were also evaluated using the Nasal polyp score (NPS) by means of a nasal endoscopy (0° and/or 30° rigid endoscope were used). Finally, at each time point, blood sample analyses were performed in order to assess hematic eosinophils’ levels.

The study was conducted in accordance with the 1996 Helsinki Declaration and was approved by each Hospital ethical committee (5304/AO/22). Informed consent was obtained from each subject before starting any study-related procedure.

### 2.2. Statistical Analysis

Quantitative variables were presented as median and interquartile range, whereas qualitative variables were expressed as number of observations and percentage. Comparisons of findings between baseline and different follow-ups were performed using the Wilcoxon test. Multiple mixed linear longitudinal regressions with a selection of variables based on Akaike’s information criterion (hybrid backward stepwise) were executed to identify the effects of the available variables on the measurement changes in time.

For all tests *p*-values have been calculated, and 5% was considered as the critical level of significance. All the analyses have been performed in R v.4.4.3 (R Core Team, Vienna, Austria, 2021) [27].

## 3. Results

A total of 217 patients (151 males and 66 females, mean age 55.8 ± 12.8 years) undergoing Dupilumab as add-on therapy for at least six months were considered for the present multicentric study. Patients’ main clinical characteristics at baseline (T0) are reported in Table 1. All patients reached at least 6 months of follow-up. One hundred and twenty-three patients reached the 12-month follow-up, while 73 patients reached the 2-year follow-up threshold (35 patients from the Padova group and 38 from the rest of the centres). Comorbid asthma was present in 68% of the population, while 61% of them suffered from inhalation allergies. The majority of the patients (87.6%) have previously undergone endoscopic sinus surgery (ESS), with a mean number of 2.4 ± 2.3 previous surgical treatments (Table 1). The mean body mass index (BMI) of the patients considered in this study was 25.9 ± 5.9 (25.8 ± 5.0 for the male group and 24.0 ± 7.4 for the female group).

NPS significantly decreased through the study period (*p* < 0.0001). In particular, NPS decreased by −2.58 (*p* < 0.001) after 6 months, by −0.65 (*p* < 0.001) between the 12th month and the 6th month of treatment and by −0.41 (*p* < 0.01) between the 24th month and the 12th month of treatment.

A similar trend is noticeable for SNOT-22, which significantly decreased throughout the initial phase of the study period (*p* < 0.0001) [in particular −37.7 (*p* < 0.001) after 6 months; −3.6 (*p* < 0.001) after 12 months], while no differences were observed between the scores collected after 24 months of treatment compared with those of the 12th month [−0.47 (*p* = 0.34)]. Additionally, all the VAS considered (NO, smell, rhinorrhea and facial pain) demonstrated a significant reduction through the study period (*p* < 0.0001 for all). When evaluating the VAS-smell in detail, it decreased by −5.27 (*p* < 0.001) after 6 months, by −0.37 (*p* < 0.001) between the 12th month and the 6th month of treatment and by −0.04 (*p* = 0.72) between the 24th month and the 12th month of treatment. Considering SSIT, there was a significant improvement in the scores measured throughout the study period (*p* < 0.0001). In greater detail, SSIT increased by 0.24 (*p* < 0.0001) in the first 6 months, while no significant differences were observed after 6 months (*p* = 0.55).

**Figure 1:**
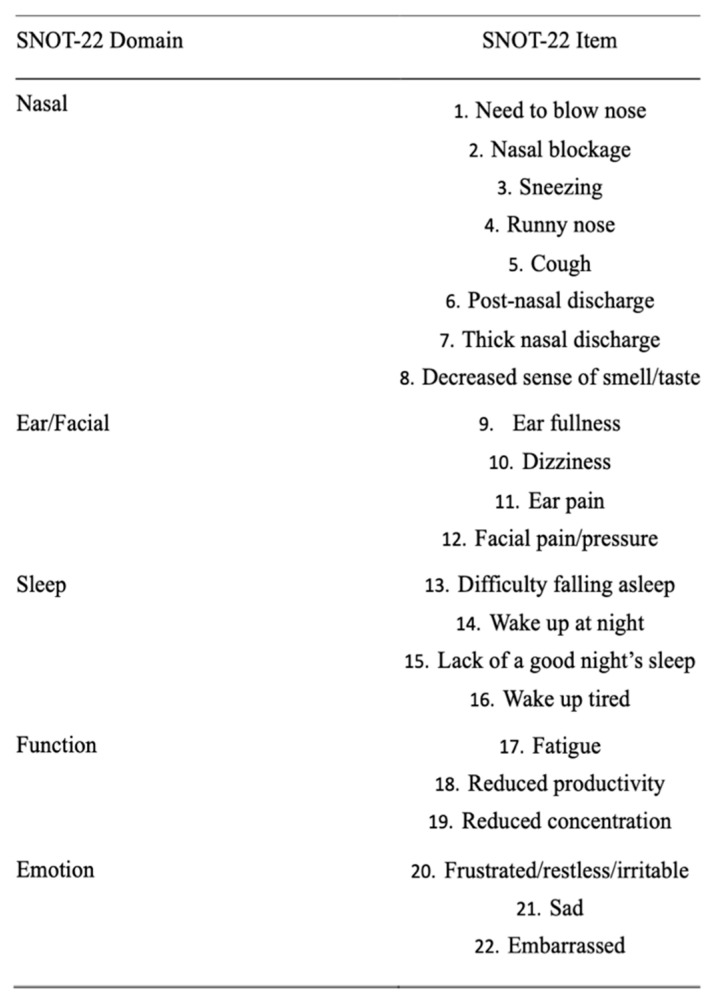
SNOT-22 items and domains.

**Figure 2:**
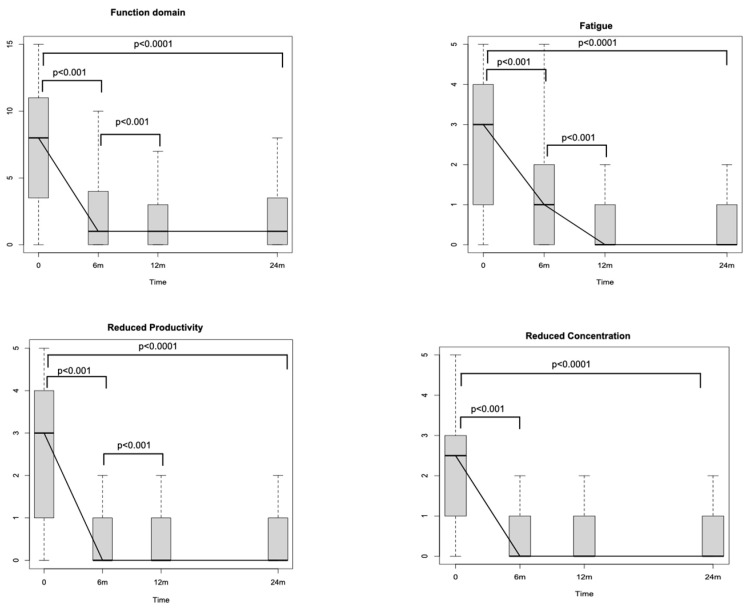
Sino-Nasal Outcome Test (SNOT-22) function domain changes during the study period. m: months.

**Figure 3:**
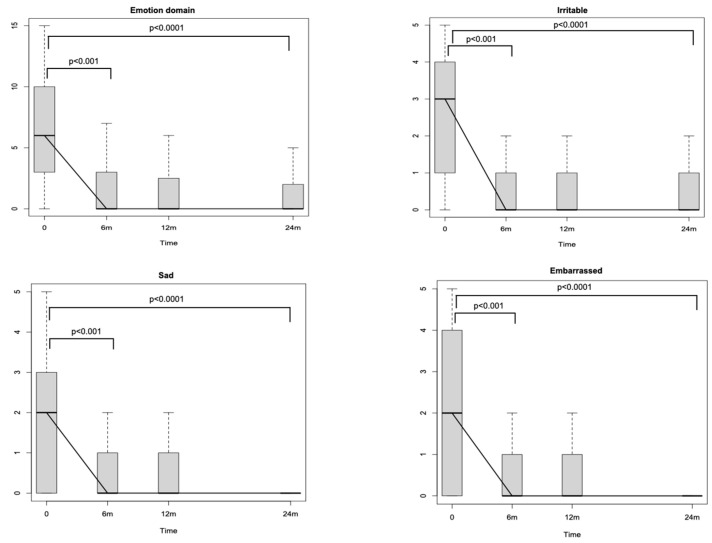
Sino-Nasal Outcome Test (SNOT-22) emotion domain changes during the study period. m: months.

Looking at SNOT-22 domains, the nasal one, which includes eight different nasal items (Figure 1), significantly decreased through the study period (*p* < 0.0001). The reductions amounted to −16.9 (*p* < 0.001) after 6 months and to −1.39 (*p* < 0.001) between T1 and T2. No differences were detected between T2 and T3 (*p* = 0.71). The ear and facial domain improved by −4.81 (*p* < 0.001) after 6 months of treatment with respect to the baseline and by −0.25 (*p* < 0.04) between T1 and T2, while no significant differences were noticed between T2 and T3 (*p* = 0.54). The sleep domain showed a score reduction of −7.02 (*p* < 0.001) between T0 and T1 and of −0.64 (*p* = 0.02) between T1 and T2. No significant differences were identified after the 12th month of treatment (between T2 and T3). The emotion domain significantly decreased after initiating Dupilumab by −4.6 between T0 and T1 (*p* < 0.001). After the sixth month, no significant differences were recognised in terms of emotion (between T1–T2 and between T2–T3). Finally, the domain of function improved between T0 and T1 [−4.74 (*p* < 0.001)] and between T1 and T2 [−0.59 (*p* < 0.001)]. No significant differences were detected between T2 and T3. Looking at the specific items within the function domain (fatigue, reduced productivity, and reduced concentration), a significant reduction through the study period of all three items is highlighted (*p* < 0.0001). In particular, each one of the three items showed a significant reduction at T1 with respect to T0 [reduced productivity −1.53 (*p* < 0.001), fatigue −1.28 (*p* < 0.001) and reduced concentration −1.46 (*p* < 0.001)], whilst only reduced productivity and fatigue showed a reduction at T2 with respect at T1 [−0.19 (*p* < 0.001) and −0.27 (*p* < 0.001), respectively] (Figure 2). Similarly, when evaluating the specific items within the emotion domain (irritable, sad and embarrassed), a significant reduction throughout the study period of all three items was detected (*p* < 0.0001). In greater detail, all three items reported a significant reduction within the first 6 months (*p* < 0.001) (T1), while no significant differences were observed thereafter (Figure 3). 

A multivariate analysis was conducted with a model including all available variables available to assess their influence on the function impairment domain. We evidenced that male sex and time significantly influenced this domain (*p* = 0.0001 and *p* = 0.000001, respectively). On the contrary, BMI had a negative impact on the domain (*p* = 0.008) (Table 2). Similar findings were outlined in the multivariate analysis when selectively considering the item-reduced productivity. There was a significant influence of male sex (*p* = 0.002) and time (*p* = 0.000001), but no influences of BMI (Table 3).

When considering the emotion domain in the multivariate analysis, male sex and time from Dupilumab initiation were shown to independently improve the domain (*p* = 0.003 and *p* = 0.000001, respectively).

Regarding the safety of Dupilumab, a total of 43 patients (19.8% of the population) experienced minor/moderate adverse events during the study period. In particular, 28/217 patients (12.9%) within the first 6 months of therapy, 12/123 patients (9.7%) between the 6th and the 12th month of therapy and 3/73 patients (4.1%) during the second year of follow-up. No major adverse events were evidenced in the population considered. The most reported adverse events included injection site dermatitis (15 patients), transient musculoskeletal pain (10 patients) and ocular symptoms such as ocular dryness/conjunctivitis (5 patients).

When looking at the blood eosinophils’ levels during the study period, the mean eosinophils’ blood count (EBC) at baseline was 0.54 ± 0.36 with 5/217 (2.3%) patients expressing hyper-eosinophilia (eosinophils > 1500 cells/mm^3^) and none with eosinophils > 3000 cells/mm^3^. At T1, mean EBC increased at 0.74 ± 0.62 cells/mm^3^, and 20/217 patients (9.2%) were found to exhibit hyper-eosinophilia (of whom 2 with eosinophils > 3000 cells/mm^3^). At T2, the mean EBC was 0.70 ± 0.61 cells/mm^3^ with 15/123 patients (12.2%) with hyper-eosinophilia (of whom 1 with eosinophils > 3000 cells/mm^3^). Ultimately, at T3, the mean EBC was 0.61 ± 0.60 cells/mm^3^, and only 4/73 patients (5.5%) had hyper-eosinophilia (of whom 1 with eosinophils > 3000 cells/mm^3^). Eleven of the twenty patients who experienced elevated eosinophil levels at T1 had persistent eosinophilia at T2, and three of them reported persistent eosinophilia also in the second year of follow-up. Only for one patient who exhibited 2 years of persistent eosinophilia (>3000 cells/mm^3^), it was decided to discontinue Dupilumab therapy despite the patient being asymptomatic. Considering the relationship between EBC and adverse events, we outlined a positive correlation at all the follow-up time points [T1 (*p* = 0.001), T2 (*p* = 0.007), T3 (*p* < 0.00001)].

Regarding Dupilumab discontinuation, 17 patients interrupted the treatment during the study period. Seven patients within the first 6 months of treatment, seven within the first year and three within the second year of treatment. The most frequent reasons for discontinuation were poor response in five patients, followed by autonomous patient interruption, despite clinical benefit and without any medical advice in 4 of these cases. After T1, three patients preferred to undergo sinus surgery, and consequently, injections were interrupted. Ultimately, Dupilumab administration was interrupted in five patients for other reasons, including the aforementioned patient with 2 years of asymptomatic persistent hyper-eosinophilia. In none of the patients who underwent Dupilumab interruption, discontinuation was due to major adverse events.

## 4. Discussion

The findings presented in this multicentric study provide valuable insights into the efficacy and safety profile of Dupilumab treatment as an add-on therapy in the management of severe and uncontrolled CRSwNP. An outstanding decrease in NPS was assessed throughout the treatment period. Despite evidencing the most substantial reduction within the 6 months of treatment, the decrease in NPS remained statistically significant at each interval, demonstrating the long-term maintenance of the positive effects of this biological therapy on nasal polyps’ shrinkage, even after 12 months of treatment. Similarly, SNOT-22 global scores have remarkably improved, with a statistically significant reduction in each interval between baseline, 6th month and 12th month, hence indicating a favourable impact on perceived disease severity, patient-reported outcomes and QoL. Correspondingly, these improvements were mirrored across all the evaluated VAS measures, reflecting a concomitant reduction in perceived NO, smell disturbances, rhinorrhea, and facial pain. Given the fact that, at the moment, no reliable type-2 inflammation biomarkers are available for CRSwNP patients [1], perhaps these results could even be more interesting. In fact, the patients recruited may also have suffered from mixed forms of inflammation, namely type 1 and type 2. In this regard, a previous experience from our group found a significant decrease of the nasal neutrophilic infiltration evaluated at nasal cytology in CRSwNP patients treated with Dupilumab [28]) meaning that in a significant percentage of CRSwNP patients, there may be a mixed inflammatory substrate.

Focusing on the olfactory function, it is worth mentioning how smell improvement was found statistically significant only within 6 months from Dupilumab’s first administration, while patients’ perception of their own olfactory function (evaluated with the VAS smell) progressively improved throughout the study period, maintaining the statistical significance between each interval between baseline, 6 months, 12 months and 24 months. These findings emphasise the divergence between olfactory perception and patients’ subjective insight into their olfactory function. Together with other previous studies, this not only demonstrates the uncertainty in the individual ability to evaluate their own smell abilities [29,30] but also highlights how the commonly used smell tests do not cover some of the relevant aspects of olfactory input perception modalities and how impactful these inputs are in the assessment of patients’ perspectives.

Remarkably, the majority of the specific SNOT-22 domains (nasal, ear/facial, sleep, and function impairment) exhibited both a score reduction in the early phases of the treatment and a statistically significant and sustained improvement of symptoms within 12 months from the baseline. This is true for all domains with the exception of the emotion one, where a statistically significant score decrease can only be observed between T0 and T1 (Figure 3) and maintains a very low score plateau thereafter, even when considering each item individually (irritable, sad, and frustrated). This pattern of reduction in the emotion domain shows how Dupilumab treatment, despite binding patients to a subcutaneous injection every 15 days, quickly induced an emotional and psychological positive effect, enhancing patients’ QoL.

Furthermore, a particular interest of this study was also to evaluate the effects of Dupilumab therapy on the SNOT-22 function domain in order to investigate its effect on patients’ production. The significant progressive improvement of the aforementioned domain during every interval within 12 months, mirrored singularly by its respective items (fatigue, reduced productivity, and reduced concentration), highlights that Dupilumab, reducing both nasal inflammation and nasal polyps’ volume, is able to enhance nasal function, i.e., olfaction, as measured by SSIT, and, eventually, patients’ productivity. This result is of paramount importance, particularly in a disease such as CRSwNP, as it is known to have significant social and economic repercussions due to poor sleep quality and loss of working days [1,31,32]. Interestingly, the multivariate analysis showed that patients’ productivity was positively and independently influenced by male sex, implying that male patients reported a stronger benefit in their productivity. This result can be linked to the fact that in Italy, workers’ occupation is higher among males (69%) than females (55%) [33]. To note, there is a gender diversity also in the Veneto region employment landscape, seeing the percentage of female workers at 59%, yet still inferior to that of males [34]. A possible explanation behind males showing higher productivity than females could be associated with the fact that at SNOT-22, males usually score lower than females, especially in the age group of our mean age population [35]. In multivariate analyses, BMI negatively influenced productivity. This influence may be more associated with the effects that BMI can have on sleep quality/performance and, consequently, on productivity [36,37]. Ultimately, both function and emotion domains were positively influenced by the time of Dupilumab therapy duration, highlighting how prolonged therapy with Dupilumab is able to remarkably reduce SNOT-22 scores related to both domains.

### 4.1. Limitations

The present study has some limitations. The first one could be the retrospective design of the study. A more important one could be that patients were treated by different ENT doctors in different ENT Units. Nevertheless, the Hospitals involved in the study comply with high-quality standards of care of patients’ evaluation and management according to ISO 9000 certification (International Organization for Standardization, Geneva, Switzerland) [38] guaranteeing a comparable practice among the different Units [1]. On the contrary, we believe that the value of a multicentric study far outweighs this possible limitation. Lastly, another possible limitation could be the lack of reliable biomarkers to identify type 2 inflammation. In this regard, there is a study showing the predictive role of CCL3 as a biochemical marker of type 2 inflammation in allergic rhinitis [39]. It certainly could be interesting in future to evaluate the role of CCL3 as a type 2 endotype biomarker in CRSwNP.

### 4.2. Safety

There is a major interest in the safety of biological drugs adopted for treating CRSwNP patients. In particular, although Dupilumab is demonstrated to be a safe drug in both phase 3 [40] and phase 4 studies [14,28], it is known that it is able to increase the absolute eosinophil count because of its mechanism of action and that, although rarely, Dupilumab induced hypereosinophilia has been associated to eosinophilic granulomatosis with polyangiitis and hypereosinophilic syndrome [41]. Importantly, no significant increase in adverse events has been observed [15]. In the present study, 43 patients (19.8% of the population) experienced adverse events during the study period. All these events were defined as minor or moderate, as all the patients with adverse events were asymptomatic or had mild symptoms, and no intervention or minimal non-invasive intervention was indicated. Most of the adverse events were injection site dermatitis and transient musculoskeletal pain, which resolved spontaneously. Focusing on the relationship between EBC and adverse events, we outlined a positive correlation at all the follow-up time points [T1 (*p* = 0.001), T2 (*p* = 0.007), T3 (*p* < 0.00001)], highlighting that higher EBC values are likely to be associated with higher, although minor, adverse events rate.

During the follow up, a small portion (at most 12%) of patients showed an increase of EBC. As already reported by previous studies [42], in most cases, this increase was transient, and the blood eosinophils count returned to the baseline values with time. In our cohort, only three patients showed persistent and asymptomatic hypereosinophilia at 2 years follow-up (4% of the population that reached T3). To the best of our knowledge, only Rampi et al. [43]. reported their results in the CRSwNP population in the second year of follow-up, with just four patients reaching the 24-month follow-up. As previously noted [44], pre-therapy EBC > 500 cells/mm^3^ is a risk factor for hypereosinophilia during the study, and our results confirm these previous observations; in fact, all of our patients affected by persistent hypereosinophilia presented EBC > 1000 cells/mm^3^ at the baseline.

Overall, no patients had to interrupt the therapy for safety reasons or major adverse events.

## 5. Conclusions

Sinonasal inflammation is associated with reduced quality of life, especially due to nasal obstruction and olfactory loss in patients affected by severe and uncontrolled CRSwNP. These symptoms, together with reduced sleep quality, are associated with a significant impact on patients’ productivity and emotions.

With the introduction of targeted biologic molecules in the management of severe CRSwNP, such as Dupilumab, patients’ QoL has substantially improved with a significant reduction of irritation, sadness and frustration and, consequently, with a long-lasting reduction of fatigue and significant improvements in both concentration and productivity.

Clinicians dealing with severe CRSwNP patients should be mindful of the emotional and productivity sphere of influence in order to have a comprehensive evaluation of the patients’ conditions.

## Figures and Tables

**Table 1:** Patients’ main clinical characteristics at baseline.

	Padova *n* = 73	Venezia Mestre *n* = 42	Cittadella *n* = 27	Rovigo *n* = 19	Schiavonia *n* = 12	Udine *n* = 10	Montebelluna *n* = 10	Treviso *n* = 8	Bassano del Grappa *n* = 8	Belluno *n* = 8	TOTAL *n* = 217
**Sex**	19 Women 54 Men	17 Women 25 Men	9 Women 18 Men	7 Women 12 Men	2 Women 10 Men	1 Women 9 Men	4 Women 6 Men	1 Women 7 Men	3 Women 5 Men	3 Women 5 Men	66 Women 151 Men
**Mean Age, yr (SD)**	52.9 (13.3)	55.3 (12.5)	57.6 (13.5)	57.2 (12.3)	62.9 (11.3)	60.1 (12.6)	55.1 (5.5)	59.5 (6.9)	56.3 (14.7)	55 (16.6)	55.8 (12.8)
**BMI**	25.9 (5.1)	23.1 (8.6)	24.6 (3.1)	N/A	28 (2.9)	N/A	N/A	26.8 (4.8)	25.7 (4.6)	26.9 (4.6)	25.3 (5.9)
**Asthma, *n* (%)**	26 (35.6)	31 (73.8)	21 (77.8)	14 (73.7)	8 (66.7)	6 (60.0)	1 (10.0)	4 (50.0)	6 (75.0)	6 (75.0)	149 (68.7)
**NSAIDs intolerance, *n* (%)**	18 (24.7)	16 (38.1)	3 (11.1)	2 (10.5)	2 (16.7)	0 (0)	5 (50.0)	1 (12.5)	4 (50.0)	4 (50.0)	63 (29.0)
**Allergy, *n* (%)**	52 (71.2)	34 (81.0)	11 (40.7)	7 (36.8)	7 (58.3)	6 (60.0)	3 (30.0)	5 (62.5)	5 (62.5)	4 (50.0)	134 (61.8)
**Smokers, *n* (%)**	9 (12.3)	5 (11.9)	3 (11.1)	NA	1 (8.3)	3 (30.0)	1 (10.0)	0 (0)	1 (12.5)	0 (0)	37 (17.1)
**Previous ESS, *n* (%)**	58 (79.5)	38 (90.5)	21 (77.8)	19 (100)	12 (100)	9 (90.0)	9 (90.0)	8 (100)	8 (100)	8 (100)	190 (87.6)
**Mean n. of previous surgeries, *n* (SD)**	1.7 (1.5)	3 (3.2)	2.9 (2.7)	2.6 (3.2)	2.1 (2.9)	2.4 (1.7)	3.4 (2.4)	2.5 (1.2)	2.9 (1.2)	3.5 (2.3)	2.4 (2.3)

SD: standard deviation; NSAIDs: non-steroidal anti-inflammatory drugs; ESS: endoscopic sinus surgery; N/A: not available.

**Table 2:** Multivariate regression model: correlations between function domain and male sex, BMI and time of Dupilumab therapy duration.

	Estimate	Standard Error	*t*-Value	*p*-Value
**Intercept**	2.116	0.303	6.992	<0.001
**Sex (M)**	−0.453	0.137	−3.308	0.001
**BMI**	0.029	0.011	2.644	0.008
**T0**	−1.304	0.081	−16.138	<0.001
**T1**	−1.554	0.089	−17.449	<0.001
**T2**	−1.537	0.112	−13.764	<0.001

M: males; BMI: body mass index; T0: Baseline; T1: 6 months of Dupilumab therapy; T2: 12 months of Dupilumab therapy.

**Table 3:** Multivariate regression model: correlations between the item-reduced productivity and male sex, BMI and time of Dupilumab therapy duration.

	Estimate	Standard Error	*z*-Value	*p*-Value
**Intercept**	0.335	0.326	1.028	0.304
**Sex (M)**	−0.427	0.136	−3.144	0.002
**BMI**	0.023	0.012	1.952	0.050
**T0**	−1.100	0.101	−10.884	<0.001
**T1**	−1.389	0.127	−10.908	<0.001
**T2**	−1.266	0.167	−7.564	<0.001

M: males; BMI: body mass index; T0: Baseline; T1: 6 months of Dupilumab therapy; T2: 12 months of Dupilumab therapy.

## Data Availability

The datasets generated and analysed during the current study are available upon reasonable request.

## Abbreviations

BMI = body mass index; CRS = chronic rhinosinusitis; CRSwNP = chronic rhinosinusitis with nasal polyps; EBC = eosinophils blood count; ESS = endoscopic sinus surgery; INCS = intranasal corticosteroids; ISO = International Organization for Standardization; M = males NO = nasal obstruction; NPS = nasal polyp score; NSAIDs = non-steroidal anti-inflammatory drugs; OCS = oral corticosteroids; QoL = quality of life; SD = standard deviation; SNOT-22 = sinonasal outcome test-22; SSIT = Sniffin’ sticks identification test; T0 = baseline; T1 = 6 months evaluation; T2 = 12 months evaluation; T3 = 24 months evaluation; VAS = Visual Analogue Scale.
